# Stated preferences in the Iranian health system: a systematic review of discrete choice experiments and conjoint analysis

**DOI:** 10.1186/s13561-026-00795-z

**Published:** 2026-05-23

**Authors:** Lucie Sabin, Mohammad Hajizadeh, Hassan Haghparast-Bidgoli, Ali Kiadaliri

**Affiliations:** 1 Institute for Global Health, University College London, London, UK; 2 School of Health Administration, Dalhousie University, Halifax, Canada; 3 Department of Clinical Sciences-Lund, Orthopedics, Lund University, Remissgatan 4, Lund, SE-221 85 Sweden; 4 Division of Insurance Medicine, Department of Clinical Neuroscience, Karolinska Institute, Stockholm, Sweden

**Keywords:** Discrete choice experiment, Stated preferences, Health, Iran, Systematic literature review

## Abstract

**Background:**

Discrete choice experiments (DCEs) are increasingly used in health to determine preferences and inform policy decisions. However, questions remain about adherence to best practices and validity of the studies. To date, no systematic review has synthesised the application of DCEs in health in Iran. We conducted a systematic review to examine characteristics, methods, quality, and topics of DCEs in Iran.

**Methods:**

We conducted a systematic search of PubMed, CINAHL, PsycINFO, Web of Science, and Scopus. Studies were included if they collected participants’ preferences for a health-related topic among relevant stakeholders using DCEs, conjoint analyses, or best-worst scaling. Data was extracted on study design, participants, attribute and choice set development, data collection, and estimation approaches. The quality of each study was assessed against good practice criteria.

**Results:**

Thirty studies met the eligibility criteria. The number of DCEs published increased from 2021 onwards. Most studies were conducted in urban areas, particularly in Tehran, the capital city, and focused on health services and workforce. Attributes were generally related to monetary, time, risk, or health outcomes. Pilot testing and qualitative work with target population to inform attribute selection and questionnaire design were conducted inconsistently. Nearly half of the studies used effective designs. However, reporting on key methodological elements such as software used, blocking, and opt-out options was often incomplete. Estimation approaches varied, with conditional logit models being most commonly used.

**Conclusions:**

This is the first systematic review of DCEs on health-related topics in Iran. Although the use of this method is expanding rapidly, gaps remain in questionnaire design, method reporting, and econometric modelling. Future DCEs would benefit from including more diverse populations, improving methodological rigour, and aligning objectives with target populations. Strengthening these aspects will improve the validity and policy relevance of DCEs in Iran.

**Supplementary Information:**

The online version contains supplementary material available at 10.1186/s13561-026-00795-z.

## Introduction

 The discrete choice experiments (DCEs) method is increasingly popular and has become widely used in health over recent decades to answer a range of questions, notably concerning how patients and providers value trade-offs in health services [1]. Originally developed by Louviere and Hensher (1982) [2] and Louviere and Woodworth (1983) [3] in the field of transport and market research, the method is grounded in Lancaster’s (1966) [4] theory that individuals obtain utility from the attributes and levels of attributes that characterise a good rather than the quantity consumed of that good. Using responses to the questionnaire, an individual’s utility function for a good can be estimated by knowing the utility provided by each attribute of that good. These values are not observed directly but are deduced from the choices individuals make.

The DCE method involves presenting participants with hypothetical choice situations to analyse the ‘utility’ of the alternatives and to assess the relative weight of the intervention’s characteristics in the trade-offs made by individuals [5]. Each hypothetical situation is described by a set of attributes, which represent different characteristics. For each attribute, the DCE provide different levels that represent different values or options. The DCE questionnaire presents combinations of these attributes and levels to participants, who then choose their preferred scenario. By systematically varying the levels of each attribute in different hypothetical scenarios, researchers determine the relative importance that individuals place on different attributes when making choices.

The growing adoption of DCEs in the health sector stems from their ability to assess the ‘non-market’ aspects of care, such as dignity, autonomy and waiting times, which are not captured by traditional clinical or financial indicators [6]. By requiring participants to make trade-offs, DCEs provide policymakers with a precise ‘marginal rate of substitution’ between different service attributes [7]. However, this method has inherent challenges [1]: it relies on the assumption of compensatory decision-making and may be subject to hypothetical bias, as stated preferences may differ from actual behaviour. Furthermore, as the number of attributes increases, the cognitive load on participants may lead them to resort to simplifying heuristics rather than deliberate choice, which can affect the reliability of utility estimates.

In Iran, as in many other countries, DCEs are increasingly being used to inform health policies and interventions in response to a growing demand for evidence-based approaches that incorporate the preferences of patients and healthcare providers. The Iranian health system is currently facing significant structural pressures, including high out-of-pocket costs for patients, regional disparities in the distribution of specialists, and economic pressures linked to international sanctions. In response to this, recent large-scale reforms [8], including the Health Transformation Plan (HTP) and the national commitment to universal health coverage (UHC) [9, 10], have necessitated a shift from a supply-driven health system to a demand-driven one. This context, marked by scarce resources and the need for prioritisation, makes DCEs particularly relevant. By enabling policymakers to accurately quantify trade-offs between service quality, cost-sharing and geographical access, DCEs provide a robust mechanism for integrating the ‘voice of the user’ to ensure the sustainability of the reforms.

Although the use of DCEs has grown in recent years, there has been no systematic review assessing the scope or methodological quality of studies conducted in Iran. By analysing how this methodology was adapted in a middle-income country during a period of far-reaching reforms, this study provided a benchmark for other countries. This analysis assessed methodological rigour and areas of application, and shed light on the transition towards health systems that are preference-driven rather than supply-driven, a key issue in the global drive towards UHC.

## Method

The review was conducted and reported in accordance with the guidelines outlined in the Preferred Reporting Items for Systematic Reviews and Meta-Analyses (PRISMA) checklist [11].

### Search strategy

On 22 February 2025, AK conducted a comprehensive search of electronic databases, including PubMed, CINAHL, PsycINFO, Web of Science, and Scopus, to identify relevant studies. The search strategy was based on the keywords and terms used in the systematic literature reviews by Bekker-Grob et al. (2012) [7]. We performed a Boolean search using combinations of terms related to ‘Discrete Choice Experiment’, ‘Conjoint Analysis’, and ‘Iran’ (see Supplementary Material 1). The finalised search terms were developed through an iterative process and adapted to the different databases. To ensure a sensitive and comprehensive capture of the literature, searches were applied to titles and abstracts.

### Study selection

The selection process was conducted on Covidence. After removing duplicates, three reviewers (AK, MH and HHB) independently checked the relevance of titles and abstracts, then assessed the full text of potentially eligible studies based on predefined inclusion criteria. Studies that met these criteria at the full-text stage were included in the final review. Any disagreements arising during the selection process were resolved by discussion.

### Inclusion criteria

Studies were included if they were original research articles that collected participants’ preferences for a health-related topic (such as a disease, treatment, health insurance scheme, or medical speciality) among relevant stakeholders (e.g., patients, clinicians, carers or health insurance managers) using DCEs, conjoint analyses, or best-worst scaling case 3 (multi-profile case). DCEs are a type of choice-based conjoint analysis, and best–worst scaling case 3 is conceptually similar to DCEs, as both methods elicit preferences by asking participants to make trade-offs between multiple attributes. Studies were excluded if they were not conducted in Iran or if they focused on non-health-related topics. Articles that were not full research papers, including conference abstracts, or that did not present original research, such as systematic reviews, were also excluded. Additionally, studies were excluded if they only identified attributes or levels of a DCE without providing a quantitative analysis of preferences, or if they were published in a language other than English.

### Data extraction

Data extraction was done on Covidence. LS used a standard form to extract key information, including study characteristics (author, year, geographic setting, area, sample size), study objectives and DCE-specific elements (attributes and levels selection process, number of attributes and levels, method to design the tasks and their number, questionnaire administration, software, analysis model).

### Quality assessment

HBB and LS assessed the quality of half of the included studies, while AK and MH evaluated the other half. Any disagreements were resolved through consensus with a third reviewer. We assessed study quality using the list of 13 validity criteria developed in a previous systematic review of DCEs [12]. That review had collated the criteria based on the comprehensive checklist proposed by Lancsar and Louviere [13] and on methodological issues highlighted in earlier DCE reviews. We selected this framework because it focuses on threats to validity, defined as the risk of bias or systematic error, across the four key stages of a DCE: conceptualisation, survey instrument development, data collection, and econometric analysis [12]. For each study, we recorded whether each criterion was met (evidence of full achievement), not met (evidence of failure to address the requirement), or partially met (where the requirement was addressed but lacked sufficient methodological rigour or detail). Finally, a criterion was recorded as unclear if the published manuscripts provided insufficient information to judge its achievement. This approach ensures that the assessment considers the quality of the presentation of results and the integrity of the underlying research methods.

### Data analysis and presentation

We assessed compliance with international standards by applying the 13 validity criteria used for the quality assessment and developed in a previous systematic review of DCEs [12]. Identifying deviations from these reference criteria enabled a standardised assessment of Iranian practices in RCTs against the global methodological consensus. Particular attention was paid to recurring themes in the literature related to DCE methodology, such as experimental design, analysis methods and validity assessment [7]. We also reviewed the thematic areas addressed by DCE studies in Iran, assessing their evolution over time and comparing them with global trends, as well as identifying the key issues addressed in these studies.

## Results

### Included studies

**Fig. 1:**
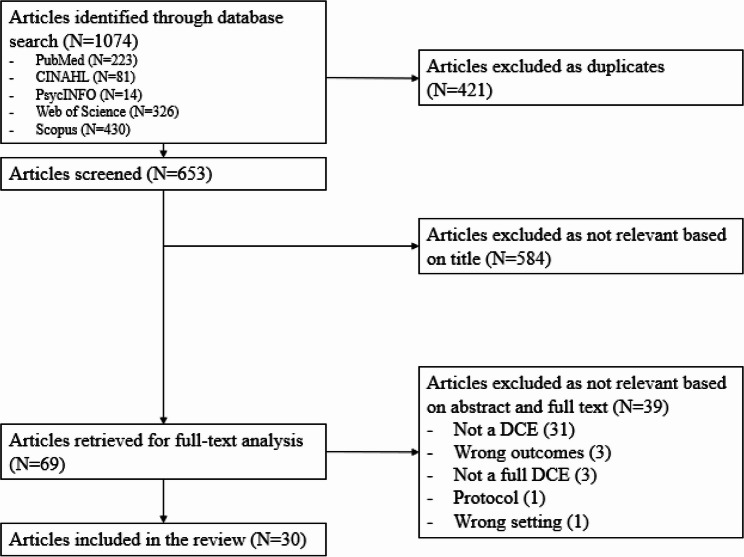
PRISMA diagram of the research strategy

After the selection process, 30 articles met the eligibility criteria and were included in the review. Supplementary Material 2 shows the full list of references and main characteristics of the included studies. The PRISMA diagram provides an overview of the selection process (Fig. 1).

### Review of included studies

The number of health-related DCEs in Iran has risen over the past decade. Whilst studies published before 2018 accounted for only 10% (*n* = 3, [14–16]), of the total literature identified, 67% (*n* = 20, [17–36]), of all studies were published from 2021 onwards. This represents an almost fivefold increase in the average annual publication rate over the last four years compared with the period before 2018. Table 1 summarises the background information of the DCE studies in Iran. Most studies were conducted in urban areas, primarily in the capital city, Tehran [14, 17, 19, 21, 23, 31, 33, 36–39]. The attributes commonly used in DCEs encompassed monetary, temporal, risk-related and health outcome attributes. Others included attributes focused on service quality and organisational factors (i.e. waiting times, staff attitudes, and facility cleanliness) and workplace-related attributes (i.e. workload and career promotion). Attributes and levels included in each study are presented in Supplementary Material 3.

In the included studies, DCEs were used to reveal the preferences of a variety of participants, mainly the general population and patients. Most of the studies included in the review focused on clinical conditions and treatment (e.g. COVID-19 vaccines and cancer treatment) [17, 19, 21, 23, 25, 29, 32, 37, 38, 40], health services (e.g. pre-hospital emergency services, quality of health services) [14, 18, 20, 24, 26, 28, 41, 42] and aspects related to the health workforce [15, 16, 22, 27, 30, 31]. Other areas of interest included health system and policy (e.g. pharmaceutical subsidy decisions, priority settings) [34, 36, 39] and health measurement and assessment [33, 35].

**Table 1:** Background information on the included studies (*N* = 30)

Item	Category	Frequency (%)
Setting	Urban	22 (73)
	Rural	1 (3)
	Both	7 (24)
Year	2010–2015	2 (6)
	2016–2020	8 (27)
	2020–2025	20 (67)
Participants	General population	11 (37)
	Patients	9 (30)
	Health workers	4 (13)
	Health-related subjects (e.g. medical students)	3 (10)
	Others	3 (10)
Topic^a^	Health service delivery	8 (27)
	Health system & policy	3 (10)
	Health workforce & human resources	7 (23)
	Clinical conditions & treatment	10 (33)
	Health measurement & valuation	2 (7)
Attributes covered^b^	Monetary	23 (77)
	Temporal	20 (67)
	Health outcome	11(37)
	Risk-related	7 (23)
	Health care	12 (40)
	Other	21 (70)

^a^Clinical conditions & treatment: studies focused on specific diseases and treatment. Health service delivery: preferences for hospital services, primary care or specific clinic-level interventions. Health workforce & human resources: job preferences, motivation, and recruitment of health workers. Health systems & policy: macro-level issues such as health insurance, financing, and system-wide reforms. Health measurement & valuation: studies developing or valuing health state utilities

^b^Totals do not add up to N as studies used many attributes

### Experimental design and choice set construction

Table 2 summarises the experimental design and construction of the choice sets in the included studies. Most studies were discrete choice experiments. Two included the composite time trade-off method [33, 35], and one was a conjoint analysis [16]. One-third of the studies did not provide a calculation method for sample size [14, 18, 28–30, 32, 34, 35, 37, 38], while another third used established formulas [16, 17, 19, 21, 25–27, 31, 36, 40–43] such as those by Cochrane [44], Orme [45], or de Bekker-Grob [46]. Approximately half of the studies [21–27, 29–31, 36, 40, 41, 43] employed an efficient design [47, 48], while 37% [15, 16, 18, 19, 32, 34, 36–39, 42] utilised a fractional factorial design [49]. Nineteen studies reported using blocks to reduce participant workload [50]. An opt-out option was available in only two studies [19, 25]. A wide range of software was used to construct the choice sets, with SAS and SPSS being the most commonly used. One-third of the studies did not report on the software used [18, 21, 25, 26, 34–38, 42], three studies (10%) did not indicate the type of experimental design [14, 33, 35], and seven (23%) did not report on the use of blocking in the design [14, 17, 18, 29, 30, 34, 35, 40, 42]. Regarding the selection of attributes and levels, most studies relied on literature review and expert consultation. On average, studies included 6 attributes and presented 11 choice tasks per respondent, with the majority presenting between 9 and 12 tasks to respondents.

**Table 2:** Experimental design and construction of choice sets (*N* = 30)

Item	Category	Frequency (%)
Study type	Discrete choice experiment	27 (90)
	Composite time trade-off method and discrete choice experiment	2 (7)
	Conjoint analysis	1 (3)
Sample size calculation	Not reported	10 (33)
	Orme’s formula	7 (23)
	Cochrane’s formula	3 (10)
	No formula, but based on literature	8 (27)
	De Bekker-Grob’s formula	2 (7)
Number of choices per respondent	8 or fewer choices	11 (37)
	9–12 choices	12 (40)
	13–16 choices	4 (13)
	More than 16 choices	3 (10)
Number of attributes	4	2 (7)
	5	9 (30)
	6	6 (20)
	7	7 (23)
	8	4 (13)
	9	2 (7)
Design type	Full factorial	1 (3)
	Fractional factorial	11 (37)
	Efficient design	14 (47)
	Other	1 (3)
	Not reported	3 (10)
Software	SPSS	6 (20)
	SAS	8 (27)
	Stata	4 (13)
	Ngene	1 (3)
	JMP	1 (3)
	Not reported	10 (33)
Use of blocks	Yes	19 (63)
	No	4 (13)
	Not reported	7 (23)
Opt-out option	Yes	2 (7)
	No	28 (93)
Attributes and levels choice^a^	Interviews	5 (17)
	Literature review	25 (83)
	Expert consultation	24 (80)
	Focus group	4 (13)
	Other	2 (7)

*JMP* Statistical software from Statistical Analysis System (SAS) Institute

^a^Totals do not add up to N as studies used different methods to determine attributes and levels

### Data collection

The data collection process varied across the included studies (Table 3). Most studies used face-to-face administered DCEs [17, 19–23, 27, 32, 33, 35–43], although online DCEs were increasingly used [16, 18, 25, 29–32, 34, 39]. Slightly more than half of the studies reported the data collection period [15, 16, 18, 20–25, 29, 31, 32, 36–40], including month and year. Pilot tests were conducted in just over half of the studies [15, 16, 21, 22, 24, 27, 31–36, 38–41]. Sample sizes varied, with the majority of studies recruiting between 200 and 500 participants. Only a few studies had fewer than 200 participants [17, 42] or more than 1,000 participants [33, 35, 36].

**Table 3:** Data collection process (*N* = 30)

Item	Category	Frequency (%)
Administration survey^a^	Self-administered questionnaire	3 (10)
	Face-to-face interview	17 (57)
	Online	7 (23)
	Phone	1 (3)
	Not reported	2 (7)
Data collection period reported (month and year)	Yes	17 (57)
	No	13 (43)
Pilot	Yes	16 (53)
	No	14 (47)
Sample size	Less than 200	3 (10)
	Between 200 and 500	16 (53)
	Between 500 and 1000	8 (27)
	More than 1000	3 (10)

^a^Totals do not add up to N as studies can use multiple methods

### Estimation procedure

Table 4 presents the estimation procedures used in the included studies. The majority of studies applied a conditional logit model, while nearly a quarter used a mixed logit model to account for heterogeneity in preferences [19, 24, 26, 30, 38, 40]. The least frequently used approaches were the probit and logit models [14, 15, 39], and generalised estimating equation models [42]. Stata was by far the most frequently used analysis software. Four studies did not indicate the software used for the analysis [17, 21, 28, 39].

**Table 4:** Estimation procedures (*N* = 30)

Item	Category	Frequency (%)
Models^a^	Conditional logit model	18 (60)
	Mixed logit model	7 (23)
	Ordered logistic model	1 (3)
	Probit model	2 (7)
	Logit model	3 (10)
	Generalised estimation equation model	1 (3)
Analysis software^b^	Not reported	4 (13)
	Stata	19 (63)
	NLogit	1 (3)
	SAS	3 (10)
	SPSS	2 (7)
	JPM	2 (7)

^b^Totals do not add up to N as studies use different estimation procedures

^b^Totals do not add up to N as some studies used more than one software

### Quality assessment

**Fig. 2:**
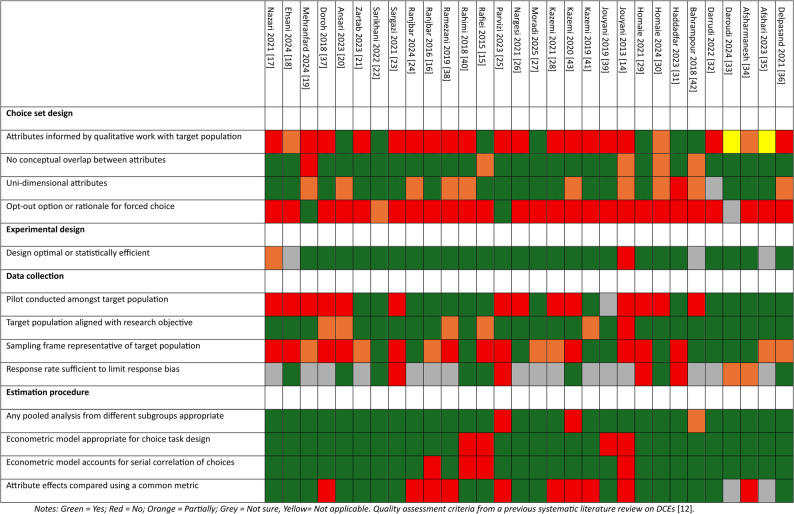
Quality assessment of the included studies (N = 30). Notes: Green = Yes; Red = No; Orange = Partially; Grey = Not sure, Yellow = Not applicable. Quality assessment criteria from a previous systematic literature review on DCEs [12]

The assessment of the quality of the included studies revealed significant heterogeneity in methodological quality (Fig. 2). Regarding the design of choice sets, only a minority of studies selected attributes based on qualitative work with the target population [15, 20, 22, 27, 29, 31, 42], and few justified the use of forced-choice models in the absence of an opt-out option [19, 25]. Most studies ensured that attributes were unidimensional and were not conceptually overlapping.

Considering experimental design, most studies employed designs described as optimal or statistically efficient. Three studies did not provide sufficient detail to fully assess the quality of the design [18, 35, 42]. In terms of data collection, over half of the studies conducted pilot tests with the target population [15, 16, 21, 22, 24, 27, 31, 32, 34–36, 38, 40, 41], and while most aligned the study population with the research objective, few provided evidence that the sample was representative [22, 24, 26, 30, 32–34, 39–42] or that response rates were sufficient to minimise bias [15, 18, 20, 22, 30, 36, 40, 43].

Estimation procedures were sometimes limited. Econometrics models were generally appropriate for the design of choice tasks, accounted for serial correlation in repeated choices, and compared the relative effects of attributes using a common measure [15, 17–23, 26, 27, 29–32, 36, 39, 40, 42].

## Discussion

This review included 30 DCEs conducted in Iran, with a noticeable increase in publications in recent years. Most studies were conducted in urban areas, particularly Tehran [14, 17, 19, 21, 23, 31, 33, 36–39], and focused on topics related to clinical conditions and treatment [17, 19, 21, 23, 25, 29, 32, 37, 38, 40], health services [14, 18, 20, 24, 26, 28, 41, 42] and workforce [15, 16, 22, 27, 30, 31]. Common attributes reflected monetary, time-related, risk-related, or health outcome dimensions. While several studies incorporated pilot testing and qualitative research to guide attribute selection and questionnaire design, these practices were applied inconsistently. Nearly half of the studies employed effective experimental designs, yet reporting on important methodological elements, such as the software used [18, 21, 25, 26, 34–38, 42], the application of blocking [14, 17, 18, 29, 30, 34, 35, 40, 42], and the inclusion of opt-out options [19, 25], was often incomplete. Conditional logit models were the most frequently used analytical approach.

The number of DCEs published increased over time, with very few studies published before 2018 [14–16] and a significant increase from 2021 onwards [17–36]. This trend is consistent with the global trend in the use of DCEs in health [51], where DCEs have become a well-established method for capturing stakeholder preferences and informing decision-makers [1]. The significant number of DCE publications from 2021 onwards in Iran likely reflects a dual influence. First, the COVID-19 pandemic has made it necessary to rapidly collect data on vaccination preferences and the resilience of healthcare workers, topics that are particularly well-suited to the DCES method. Second, this period coincided with the maturation of the Iranian Health Transformation Plan (HTP), which heightened the demand for patient-centred evidence to evaluate hospital performance and insurance reforms [52].

The review suggested that most DCEs were conducted in urban areas, mostly in Tehran [14, 17, 19, 21, 23, 31, 33, 36–39]. While this concentration can be explained by easier access to participants, it may also limit the externality of the results and their generalisability to rural populations, whose preferences may differ significantly due to variations in access to care, socioeconomic conditions, and health needs [53, 54]. The international literature also showed a predominance of urban settings in DCEs. Nonetheless, there has recently been a trend toward extending studies to rural and underserved populations to better capture the heterogeneity of preferences.

The thematic areas covered by the included DCEs varied widely. Similar to findings in other low- and middle-income countries [7], this diversity demonstrated that DCEs can be applied to a wide range of priority areas. The focus on health services and workforce issues in DCEs conducted in Iran aligned with the ongoing reforms in the country that aimed at strengthening service delivery and addressing human resource challenges in the health sector [55]. In line with this diversity of topics, the types of respondents also varied considerably, ranging from patients and the general population to healthcare workers and students. While this variation highlighted the versatility of DCEs, it also underscored the importance of ensuring that the population studied is well-suited to the specific objective of the research. Since response rates were not reported in most of the studies, it was difficult to assess representativeness and risk of bias.

In terms of attributes, the most used categories were monetary, time-related, risk-related, and health outcome-related attributes. This is consistent with international practice, where these attributes are frequently considered to quantify trade-offs [7]. Most of the DCEs included reported basing their attributes on literature reviews and qualitative works. However, most of the qualitative investigations were not conducted with the targeted population, as recommended by best practices to ensure the relevance and validity of attributes [13, 56]. For example, several studies aiming at eliciting patients’ preferences, selected attributes using literature review and interviews with physicians or experts only [14, 28]. While most of the included DCEs met standards, others did not provide sufficient detail, limiting the assessment of attribute quality.

Regarding experimental design, most studies employed efficient designs [21–27, 29–31, 36, 40, 41, 43] or fractional factorial designs [15, 16, 18, 19, 32, 34, 36–39, 42]. These models are consistent with global practice [7], where efficient designs are preferred to balance statistical precision and respondent burden [57]. The frequent use of blocking in the included studies also reflects efforts to reduce cognitive load.

Data collection methods varied, with most studies relying on face-to-face administration of the DCE [17, 19–23, 27, 32, 33, 35–43]. However, online administration accounted for 30% of the studies included [16, 18, 25, 29–32, 34, 39], and the trend is increasing over the years. The growth of online administration is consistent with international trends [12]. Online surveys offer advantages in terms of reduced costs, faster data collection and the ability to reach large and geographically dispersed populations [58]. Though, they also raise important questions about their representativeness, as access to and familiarity with digital technologies vary considerably across socio-economic and geographical groups [59]. In Iran, internet access remains uneven, with 69.2% of households with internet access at home in rural areas compared to 82.5% in urban areas [60]. This disparity suggests that the use of online surveys may reinforce the existing urban bias observed in the included studies.

Pilot testing was conducted in just over half of the studies [15, 16, 21, 22, 24, 27, 31–36, 38–41], indicating variability in adherence to best practices [13]. In Iran, where health literacy levels [61], familiarity with survey research, and access to health information [62] vary across regions and social groups, pilot testing is particularly important. Without pilot testing, participants may misunderstand the scenarios presented, leading to unreliable or biased preference estimates [13].

Estimation procedures were dominated by conditional logit models, with a small proportion of included studies using mixed logit models [19, 24, 26, 30, 38, 40]. Although conditional logit models remain widely used internationally, some evidence suggests that they may produce biased estimates in the presence of preference heterogeneity [63]. Good practice guidelines recommend taking this heterogeneity into account. However, there is still no consensus on the most appropriate modelling approach to achieve this [64]. Furthermore, half of the studies did not compare the relative effects of attributes using a common measure, which complicates interpretation across attributes.

In describing the method used in the included studies, several shortcomings were identified. One-third of the studies did not specify the software used for the design of the questionnaire [18, 21, 25, 26, 34, 35, 36, 37, 38, 42], and some studies did not mention the type of experimental design used [14, 33, 35] or the use of blocking [14, 17, 18, 29, 30, 34, 35, 40, 42]. These omissions are not in line with the reporting guidelines for DCEs [65], yet transparency in reporting is essential for assessing the quality and reproducibility of studies.

Despite the utility of DCEs in informing Iranian health policy, their transition into practical application remains nascent. To bridge the ‘know-do’ gap and ensure that DCEs serve as credible evidence for decision-makers, future DCEs conducted in Iran should involve policy-makers in attribute selection to ensure the trade-offs studied align with current legislative agendas, adhere to reporting standards to build trust in the evidence’s validity and prioritise rural and underserved populations to ensure equitable policy outcomes. Without these elements, DCEs risk remaining academic exercises rather than transformative policy tools.

This review is subject to some limitations. First, due to resource and time constraints, the review was limited to studies published in English, which may have included a publication bias by excluding relevant publications in Persian, the official language of Iran. Second, although an exhaustive search was conducted in the main databases, unpublished studies and grey literature were not considered. Finally, while the review assessed methodological practices, the quality of the studies could not always be evaluated from the manuscripts published.

## Conclusion

Overall, the findings suggest that DCEs are being used increasingly in Iran. However, notable gaps remain in reporting, attributes development, and analysing data, particularly given the complexity of choices. These gaps were consistent with those identified in systematic reviews of DCEs conducted worldwide [1, 7, 12], where transparency and methodological rigour remain uneven despite the existence of checklists and best practice guidelines [66–68].

To increase their impact, future DCEs conducted in Iran should extend beyond urban and Tehran-based populations to include rural and marginalised groups, thereby improving the generalisability and policy relevance of the results. Qualitative work directly involving the targeted population should be strengthened to inform choices about attributes and levels. Reporting on design and analytical choices should also be improved in line with international checklists [66–68], as well as the broader adoption of econometrics models that allow for the exploration of preference heterogeneity [64].

## Supplementary Information

Supplementary Material 1.

## Data Availability

No datasets were generated or analysed during the current study.

## Abbreviations

DCE: Discrete Choice Experiment
PRISMA: Preferred Reporting Items for Systematic Reviews and Meta-Analyses
