# Supplementary material for: Stated preferences in the Iranian health system: a systematic review of discrete choice experiments and conjoint analysis
Source: Health Econ Rev. 2026 May 23;16:68. doi: 10.1186/s13561-026-00795-z (PMC13217655; doi:10.1186/s13561-026-00795-z)
Supplement: Supplementary file 1 — Supplementary Material 1. [file 13561_2026_795_MOESM1_ESM.docx]

**Supplementary Material 1 – Final Search Term**

PubMed: "discrete choice" OR "choice experiment*" OR "choice model*" OR DCE OR "conjoint analysis" OR "conjoint experiment*" OR "choice?based conjoint" OR "conjoint stud*" OR "stated choice*" OR "stated preference*" OR "factorial survey*" OR "part-worth utilities" OR "paired comparison*" OR "pairwise choice*" OR "discrete-choice" OR "preference elicitation" OR "best-worst" OR "maxdiff" AND (iran) OR (iran[MeSH Terms])

Cinhal: TX ("discrete choice" OR "choice experiment*" OR "choice model*" OR DCE OR "conjoint analysis" OR "conjoint experiment*" OR "choice?based conjoint" OR "conjoint stud*" OR "stated choice*" OR "stated preference*" OR "factorial survey*" OR "part-worth utilities" OR "paired comparison*" OR "pairwise choice*" OR "discrete-choice" OR "preference elicitation" OR "best-worst" OR "maxdiff") AND (MW iran OR MH iran OR MJ iran OR MM iran OR DH iran OR TI iran OR AB iran OR SU iran)

Psycoinfo: TX ("discrete choice" OR "choice experiment*" OR "choice model*" OR DCE OR "conjoint analysis" OR "conjoint experiment*" OR "choice?based conjoint" OR "conjoint stud*" OR "stated choice*" OR "stated preference*" OR "factorial survey*" OR "part-worth utilities" OR "paired comparison*" OR "pairwise choice*" OR "discrete-choice" OR "preference elicitation" OR "best-worst" OR "maxdiff") AND (MW iran OR MH iran OR MJ iran OR MM iran OR DH iran OR TI iran OR AB iran OR SU iran)

Web of Sciences: ("discrete choice" OR "choice experiment*" OR "choice model*" OR DCE OR "conjoint analysis" OR "conjoint experiment*" OR "choice?based conjoint" OR "conjoint stud*" OR "stated choice*" OR "stated preference*" OR "factorial survey*" OR "part-worth utilities" OR "paired comparison*" OR "pairwise choice*" OR "discrete-choice" OR "preference elicitation" OR "best-worst" OR "maxdiff") AND (iran (Topic) OR iran (Title) or iran (Abstract) or iran (author keywords))

Scopus: (TITLE-ABS-KEY ("discrete choice" OR "choice experiment*" OR "choice model*" OR DCE OR "conjoint analysis" OR "conjoint experiment*" OR "choice?based conjoint" OR "conjoint stud*" OR "stated choice*" OR "stated preference*" OR "factorial survey*" OR "part-worth utilities" OR "paired comparison*" OR "pairwise choice*" OR "discrete-choice" OR "preference elicitation" OR "best-worst" OR "maxdiff") AND (TITLE-ABS-KEY (iran))

**Supplementary Material 2 – Extraction Table**

|  | **Background** | |  | **Experimental design** | | | | |  | **Data collection** | | | | | **Estimation procedure** | | |  |
| --- | --- | --- | --- | --- | --- | --- | --- | --- | --- | --- | --- | --- | --- | --- | --- | --- | --- | --- |
| **Author-date** | **Topic** | **Province** | **Method** | **Attributes** | **Design** | **Software** | **Blocks** | **Tasks** | **Sample calculation** | **Opt-out** | **Date** | **Sample** | **Pilot** | **Admin** | **Model** | **Software** | **Results reported** | |
| Nazari 2021 [1] | Clinical conditions & treatment | Tehran | DCE | 5 | Other | SPSS | NR | 16 | Orme's formula | No | No | 78 | No | Face-to-face | Conditional logit | NR | Coefficients | |
| Ehsani 2024 [2] | Health service delivery | Guilan | DCE | 6 | Fractional factorial | NR | NR | 13 | Not reported | No | Yes | 492 | No | Online | Conditional logit | Stata | Coefficients, WTP | |
| Mehranfard 2024 [3] | Clinical conditions & treatment | Tehran | DCE | 5 | Fractional factorial | SAS | No | 11 | Cochran formula | Yes | No | 384 | No | Face-to-face | Mixed logit | Stata, NLogit | Odds ratios, WTP | |
| Doroh 2018 [4] | Clinical conditions & treatment | Tehran | DCE | 9 | Fractional factorial | NR | Yes | 7 | Not reported | No | Yes | 500 | No | Face-to-face | Conditional logit | SAS | Odds ratios | |
| Ansari 2023 [5] | Health service delivery | Qazvin | DCE | 7 | Full factorial | SPSS | Yes | 9 | Based on the literature | No | Yes | 375 | No | Face-to-face | Probit, logit | Stata | Odds ratios, choice probabilities, WTP | |
| Zartab 2023 [6] | Clinical conditions & treatment | Tehran | DCE | 7 | Efficient design | NR | Yes | 8 | Orme’s and Cochrane’s fomula | No | Yes | 306 | Yes | Face-to-face | Conditional logit | NR | Odds ratios, WTP | |
| Sarikhani 2022 [7] | Health workforce & human resources | Tehran, Fars, Khorasan-Razavi, Kerman, Kermanshah, Khuzestan | DCE | 8 | Efficient design | SAS | Yes | 9 | Based on literature | No | Yes | 720 | Yes | Face-to-face | Conditional logit | Stata | WTP | |
| Sargazi 2021 [8] | Clinical conditions & treatment | Tehran | DCE | 5 | Efficient design | SAS | Yes | 9 | Based on literature | No | Yes | 306 | No | Face-to-face | Conditional logit | SAS | Coefficients | |
| Ranjbar 2024 [9] | Health service delivery | Yazd | DCE | 6 | Efficient design | SAS | Yes | 8 | Based on literature | No | Yes | 301 | Yes | Paper | Mixed logit | Stata | Odds ratios | |
| Ranjbar 2016 [10] | Health workforce & human resources | All over Iran | Conjoint analysis | 7 | Fractional factorial | SPSS | No | 18 | Orme's formula | No | Yes | 350 | Yes | Online | Ordered logistic | Stata | Odds ratios | |
| Ramezani 2019 [11] | Clinical conditions & treatment | Tehran | DCE | 9 | Fractional factorial | NR | Yes | 7 | Not reported | No | Yes | 500 | Yes | Face-to-face | Mixed logit | SAS | Odds ratios | |
| Rahimi 2018 [12] | Clinical conditions & treatment | Isfahan | DCE | 6 | Efficient design | JMP | NR | 12 | Cochran formula | No | Yes | 358 | Yes | Face-to-face | Mixed logit | SPSS | Relative attribute importance | |
| Rafiei 2015 [13] | Health workforce & human resources | 5 provinces | DCE | 8 | Fractional factorial | SPSS | No | 15 | Based on literature | No | Yes | 120 | Yes | NR | Probit | Stata | Marginal effects, WTP | |
| Parvizi 2023 [14] | Clinical conditions & treatment | All over Iran | DCE | 7 | Efficient design | NR | Yes | 12 | Orme's formula | Yes | Yes | 678 | No | Online | Conditional and mixed logit | Stata | Odds ratios | |
| Nargesi 2021 [15] | Health service delivery | All over Iran | DCE | 5 | Efficient design | NR | No | 12 | De Bekker-Grob's formula | No | No | 811 | No | Phone | Mixed logit | Stata | WTP, marginal rates of substitution | |
| Moradi 2025 [16] | Health workforce & human resources | Kermanshah | DCE | 6 | Efficient design | Stata | Yes | 6 | De Bekker-Grob's formula | No | No | 500 | Yes | Face-to-face | Conditional logit | Stata | Odds ratios, WTP | |
| Kazemi 2021 [17] | Health service delivery | Kermanshah | DCE | 5 | Efficient design | NR | Yes | 7 | Not reported | No | No | 328 | No | Face-to-face | Conditional logit | NR | Odds ratios | |
| Kazemi 2020 [18] | Health workforce & human resources | Kermanshah | DCE | 7 | Efficient design | SAS | Yes | 8 | Orme's formula | No | No | 201 | No | Face-to-face | Conditional logit | Stata | Odds ratios | |
| Kazemi 2019 [19] | Health service delivery | Tehran | DCE | 8 | Efficient design | SAS | Yes | 8 | Orme's formula | No | No | 600 | Yes | Face-to-face | Conditional logit | Stata | Odds ratios | |
| Jouyani 2019 [20] | Health system & policy | Tehran | DCE | 5 | Fractional factorial | SPSS | Yes | 18 | Based on literature | No | Yes | 579 | Yes | Face-to-face, online | Logit | NR | Odds ratios | |
| Jouyani 2013 [21] | Health service delivery | Tehran | DCE | 5 | NR | SPSS | NR | 10 | Not reported | No | No | 330 | No | Not reporter | Logit | Stata | Coefficients | |
| Homaie 2021 [22] | Clinical conditions & treatment | All over Iran | DCE | 4 | Efficient design | Stata | NR | 14 | Not reported | No | Yes | 617 | No | Online | Conditional logit | Stata | WTP, relative attribute importance | |
| Homaie 2024 [23] | Health workforce & human resources | Guilan | DCE | 7 | Efficient design | Stata | NR | 18 | Not reported | No | No | 317 | No | Online | Mixed logit | Stata | Coefficients, willingness to accept | |
| Haddadfar 2023 [24] | Health workforce & human resources | Tehran | DCE | 8 | Efficient design | SAS | Yes | 7 | Johnson and Orme's fomula | No | Yes | 243 | Yes | Online | Conditional logit | JPM | Willingness-to-pay, predicted choice probabilities | |
| Bahrampour 2018 [25] | Health service delivery | Kerman | DCE | 6 | Fractional factorial | NR | NR | 12 | Peduzzi’s formula | No | No | 167 | No | Face-to-face | Generalised eq. | Stata | Marginal effects, attribute prioritisation | |
| Darrudi 2022 [26] | Clinical conditions & treatment | Tehran, Khorasan Razavi, Isfahan, Alborz | DCE | 5 | Fractional factorial | Stata | Yes | 9 | Not reported | No | Yes | 715 | Yes | Face-to-face, online | Conditional logit | Stata | WTP, relative attribute importance | |
| Daroudi 2024 [26] | Health measurement & valuation | Tehran | Composite time trade-off method and DCE | 6 | NR | Ngene | Yes | 10 | Based on the literature | No | No | 3061 | Yes | Face-to-face | Conditional logit | Stata | Value sets, relative attribute importance | |
| Afsharmanesh 2021 [27] | Health system & policy | All over Iran | DCE | 4 | Fractional factorial | NR | Yes | 10 | Not reported | No | No | 544 | Yes | Online | Conditional logit | JPM | Relative attribute importance, choice probabilities | |
| Afshari 2023 [28] | Health measurement & valuation | Tehran, Tabriz, Mashhad, Kerman, Yazd | Composite time trade-off method and DCE | 5 | NR | NR | Yes | 7 | Not reported | No | No | 1004 | Yes | Face-to-face | Conditional logit | Stata | Value sets, relative attribute importance | |
| Delpasand 2021 [29] | Health system & policy | Tehran | DCE | 7 | Fractional factorial | SAS | Yes | 8 | Cochran formula | No | Yes | 1046 | Yes | Face-to-face | Conditional logit | SPSS | Coefficients, relative attribute importance | |

Notes: NR=Not reported, DCE=Discrete choice experiment, Admin= Administration, WTP=willingness-to-pay

**Supplementary Material 3 – Attributes and levels**

| Author-date | Topic | Attribute 1 | Attribute 2 | Attribute 3 | Attribute 4 | Attribute 5 | Attribute 6 | Attribute 7 | Attribute 8 | Attribute 9 |
| --- | --- | --- | --- | --- | --- | --- | --- | --- | --- | --- |
| Afshari 2023 | Health measurement | Mobility (no problem, slight, moderate, severe, unable) | Self-care (no problem, slight, moderate, severe, unable) | Usual activities (no problem, slight, moderate, severe, unable/extreme) | Pain/discomfort (no problem, slight, moderate, severe, extreme) | Anxiety (no problem, slight, moderate, severe, extreme) |  |  |  |  |
| Afsharmanesh 2021 | Health system | The severity of disease without treatment (Moderate, severe, without changing in quality of life) | Health gain after treatment (Relative health, full health) | Prevalence of the disease (Rare, not rare) | Cost of treatment per patient for a month (Less than 1000,000 Rial; 100,000–2000,000 Rial; 2000,000–5000,000 Rial; more than 5000,000 Rial |  |  |  |  |  |
| Ansari 2023 | Health services | Physician communication (good, moderate, poor) | Nursing communication (good, moderate, poor) | waiting time (less than a week, one week to one month and more than a month) | service continuity (yes, no) | complimentary insurance coverage (yes, no) | hoteling services quality (good, poor) | treatment tariff (1.5 times the government tariff, 1.5–twice the government tariff and three times the government tariff) |  |  |
| Bahrampour 2018 | Health services | Waiting time for admission (half an hour, two hours, five hours) | Medical staff attention to patients at hospital (high, average, low) | Physical examination quality (high qulity, low quality) | Staff attitude (friendly, indifferent) | Cleanliness (frequently clean, sometimes clean) | After discharging training (done, not done) |  |  |  |
| Daroudi 2024 | Health measurement | Physical functioning (level 1 to 6) | Role limitations (level 1 to 6) | Social functioning (level 1 to 6) | Pain (level 1 to 6) | Mental health (level 1 to 6) | Vitality (level 1 to 6) |  |  |  |
| Darrudi 2022 | Covid | Effectiveness (50%, 70%, 90%) | Risk of severe side effects (1 person per one million people, 5 people, 10 people) | Price (free, 500 thousand rials, 1 million rials, 2 million rials, 5 million rials) | Location of vaccine production (imported, domestic production) | Protection period (6 months, 12 months, 24 months) |  |  |  |  |
| Delpasand 2021 | Health system | Increasing survival after treatment (no effect, 1 year, 5 years, 10 years) | Promoting quality of life after treatment (no effect, low improvement, average improvement, high improvement) | Alternative treatment (yes, no) | Age group of the target population (less than 18 years, 18 to 60 years, over 60 years, all age) | Cost burden for the government (10 million IRR, 100 million IRR, 500 million IRR) | Disease severity (15 years longevity and 60% QoL, 15 years longevity and 30% QoL, up to 3 months longevity and 30% QoL, | Drug manufacturer country (domestic production, imported) |  |  |
| Doroh 2018 | Cancer | Process (1 stool sample, 2 stool, 3 stool, A flexible tube that has a little camera is inserted into your rectum and through your colon; the test do not has sedation, A flexible tube that has a little camera is inserted into your rectum and through your colon, the test has sedation, Air and a white liquid are injected into your colon through a tube that is inserted into your rectum and through your colon and as the liquid moves through your colon X - rays are taken) | Pain (little pain, no pain) | Place (home, hospital) | Frequency (every year, every 5 years, every 10 years) | Preparation (no preparation, avoid certain foods and medications, laxative, enema) | Mortality/reduction (mortality risk won't change, 10 out of 100 people can survive, 40 out of 100 people can survive) | Sensitivity (3 out of 10 people with cancer, 5 out of 10 people with cancer, 7 out of 10 people with cancer) | Complication risk (No one, 1 out of 1000 people, 10 out of 1000 people) | Cost (800000 Rials, 2000000 Rials, 4000000 Rials) |
| Ehsani-Chimeh 2024 | Health services | Ambulance type (public, private) | Transfer fee (1 million, 3 million, 5 million, 10 million rials) | Time until the ambulance arrives (3, 6, 10 & 15 minutes) | Time to reach the hospital (3, 6, 10 & 15 minutes) | Type of equipment ( Essential equipment (basic necessities for an ambulance), normal equipment (standard equipment required in an ambulance), and complementary equipment (additional helpful but non-essential items)) | Emergency medical services personnel (emergency medical services technician, nurse) |  |  |  |
| Haddadfar 2023 | Health workforce | Salary (100 million IRR (250 USD) 150 million IRR (375 USD) 200 million IRR (500 USD)) | Location (city, rural) | Type of employment contract (Gharardadi, Peymani, Rasmi) | Workload (heavy, moderate, low) | Type of health facility (hospital, clinic, health house) | Workplace facilities (adequate, inadequate) | Work schedule (appropriate, inappropriate) | Expected time spent on the assigned job for promotion to a higher position (1 to 3 years, 3 to 5 years, more than 5 years) |  |
| HomaieRad 2021 | Covid | Salary (US$217, US$379, US$518, US$811, US$1081) | Contract duration (14 months, 16 months, 18 months, 24 months) | Workplace remoteness (Nonresidence and far, nonresidence but neighbor and near, nonresidence and neighbor, the province of residence, the city of residence) | Work difficulty (easy, average, difficult) | Development status of the workplace (high-developed, semi-developed, less-developed) | Welfare amenities (none, transportation, home, home and transportation) | Location of work (city, rural areas) |  |  |
| HomaieRad 2024 | Health workforce | Number of days being isolated (0, 1 week, 2 weeks, 3 weeks, one month) | Payment of being isolate (0, One-quarter of household income, Half of household income, Three-quarter of household income, Equal to household income) | COVID-19 treatment payments (0, One-quarter of the total treatment cost, Half of the total treatment cost, Three-quarter of the total treatment cost, Total treatment cost) | Services (No services, Food and essential services, Food and essential services and entertainments) |  |  |  |  |  |
| Jouyani 2013 | Health services | Waiting time from arrival until hospital admission (half an hour, two hours, five hours) | Handling of patients by medical staff at the hospital (high, average, low) | Examination type (full examination, incomplete examination) | Personnel behavior (friendly, indifferent) | Sector and doilets cleaning (frequently cleaning, sometimes cleaning) |  |  |  |  |
| Jouyani 2019 | Health system | Level of emergency (elective admission, urgent admission, emergency admission) | Severity of disease (30% health lose, 30%-70% health lose, >70% health, lose) | Communicable (yes, no) | Benefit from treatment (30% health gain, 30%-70% health gain, >70% health gain) | Age (<15, 15-65, >65) |  |  |  |  |
| KazemiKaryani 2019 | Health services | Public hospital benefits (coverage of 60/90% of costs) | Private hospital benefits (coverage of 50/70/90% of costs) | Outpatient benefits (coverage of 50/70/90% of costs) | Dental care coverage benefits (coverage of 40/70% of costs) | Rehabilitation and para-clinical benefits (coverage of 50/70/90% of costs) | Long-term care benefits (coverage of 50/70/90% of costs) | Medical devices benefits (coverage of 60/90% of costs) | Premium (250 000,350 000,450 000 Rials) |  |
| KazemiKaryani 2020 | Health workforce | Payment (capitation/capitation +30% bonus/capitation +50% bonus) | Place of medical center (own province/a nearby province/a faraway province) | Financial settlements (max to 15 days/between 15 and 30 days/ between 30 and 60 days) | Housing and transportation facilities (no/yes) | Duration of contract (one year/3 years/5 years) | Quotations for continuing education (no/after 4 years’ work in deprived area(s)/after 8 years’ work in deprived area(s)) | Workload (low/moderate/heavy) |  |  |
| Kazemi-Karyani 2021 | Health services | Waiting time for admission (1 hour, 2 hours, 3 hours and more) | Quality of physician care (Low (physician has not a friendly approach with patient. Unlikely he/she provides the patient with the necessary information about the disease, diagnostic tests and treatment), moderate (physi‑ cian has a friendly approach with patient. He/she provides the patient with the necessary informa‑ tion about the disease, diagnostic tests and treatment. He/she might have any other good qualities), good (physician has a friendly approach with patient. He/she provides the patient with the necessary information about the disease, diagnos‑ tic tests and treatment. He/she also involves the patient in making decisions)) | Quality of nursing care (Low (nurses have not a friendly approach with patient. Unlikely, they provide patient with under‑ standable information about the patient care), moderate (nurses have a friendly approach with patient. They provide patient with understandable information about the patient care), good (nurses have a friendly approach with patient. They provide patient with understandable information about the patient care and reassurance for the patient. they also involves the patient in making decisions)) | Cleaning of wards and toilets(Often clean, almost clean, always clean) | Behavior of staff (Indifferent, friendly) |  |  |  |  |
| Mehranfard 2024 | Covid | Mental illness (low, medium and high) | Unemployment (low, medium and high) | Changes in social activities (low, medium and high) | Family problems (low, medium and high) | Concern about social discrimination (low, medium and high) | Price (10,000,000 Iranian Rial, 40,000,000 Iranian Rial and 80,000,000 Iranian Rial). |  |  |  |
| Moradi 2025 | Health workforce | Monthly salary (20% decrease in current salary, 10% decrease in current salary, Current salary, 10% increase in current salary, 20% increase in current salary) | Commute time (15 min, 30 min, 45 min) | Workplace facilities (poor, average, good) | Workload (low, moderate, heavy) | Type of employment contract (Rasmi, Paymani, Gharardadi) | Time required for promotion to a higher position (1 to 3 years, 3 to 5 years, more than 5 years) |  |  |  |
| Nargesi 2021 | Health services | Price (free, 0.66US$, 1.33US$, 3.33US$) | Experience (2 years, 5 years, 10 years, 20 years) | Time (immediately, 15 min, 30 min, 60 min) | Physician (General physician, emergency medicine specialist) | Responsibility (low, middle, high) |  |  |  |  |
| Nazari 2021 | Cancer | Progression-free survival (13 months, 19 months, 24 months) | Stomatitis, grade II & III (yes, no) | Neutropenia, grade III & IV (yes, no) | Arthralgia, grade III & IV (yes, no) | Administration mode (daily-oral, monthly-muscular) | Monthly cost (100 €, 500€, 900€) |  |  |  |
| Parvizi 2023 | Covid | Effectiveness (60%, 80%, 95%, 99%) | Risk of severe side effects (1/1000000, 10/1000000, 100/1000000) | Risk of mild side effects (1/10, 3/10, 5/10) | Number of doses (one dose, two doses, more than 2 doses) | Duration of protection (6 months, 12 months, lifetime) | Location of manufacture (import product, domestic product) | Price (free, 2000000 IIR, 4000000 IIR, 6000000 IIR) |  |  |
| Rafiei 2015 | Health workforce | Location (urban, rural) | Income (2000$, 4000$, 5000$, 6000$) | Dual practice (not permitted, permitted) | Workload (Low, moderate, high) | Family proximity (near, far) | Educational facilities (basic, superior) | Clinical infrastructure (inadequate, adequate) | Housing (none, basic, superior) |  |
| Rahimi 2018 | Medicines | Manufacturing country (Iran, others) | Monthly costs (0-33$, 33-132$, 132-231$) | Administration and frequency (muscular -once a week, Subcutaneously- 3times a week 0.218 0.0740, Subcutaneously-every other day) | Effectiveness (moderate, high) | Side effects (low, moderate) | Ease of injection (easy, hard) |  |  |  |
| RamezaniDoroh 2019 | Cancer | Physical functioning (level 1 to 6) | Role limitations (level 1 to 6) | Social functioning (level 1 to 6) | Pain (level 1 to 6) | Mental health (level 1 to 6) | Vitality (level 1 to 6) |  |  |  |
| Ranjbar 2024 | Health services | Contract duration (1 year, 3 years, 5 years) | Payment mechanism (capitation, capitation +15% bonus, capitation +25% fee for service) | Contract employer (health insurance, university of medical sciences, medical council) | Individuals covered (1500 people, 2400 people, 4000 people) | Catchment area (limited range to 1,5 km around GP's office, range between 1,5 to 5 km around GP's office, no geographical limitation) | Right to provide services outside of specificied package (no, yes) | Benefits such as quota for admission to specilisation courses (no benefit, after 5 years as a family physician, after 10 years as a family physician) |  |  |
| RanjbarEzatabadi 2016 | Health workforce | Waiting time (60 min, 90 min, 120 min) | Quality of care (moderate, good) | Travel time (10 min, 20 min, 30 min) | Hospital type (public, private, social security) | Provider competency (moderate, good, excellent) | Hospital facilities (poor, moderate, full) |  |  |  |
| Sargazi 2021 | Medicines | Protection against cervical cancer (50%, 70%, 90%) | Protection against genital warts (0%, 90%) | Protection duration (6 years, 25 years, 100 years) | Serious side effects (1:750,000, 1:150.000, 1:30,000) | Cost (0US$, 95US$, 167US$) |  |  |  |  |
| Sarikhani 2022 | Health workforce | Expected monthly income (200 million IRR, 300 million IRR, 500 million IRR, 700 million IRR) | Opportunity for procedural activity (great, ordinary, rare) | Work-family compatibility (high, relative, low) | Opportunity for academic careers (great, ordinary, undesirable) | Occupational prestige (excellent, ordinary) | Job burnout (high, medium, low, very low) | Emergency or on-call schedule (often, sometimes, seldom) | Scope of practice (broad, narrow, constant) |  |
| Zartab 2023 | Medicines | Route of administration (Subcutaneous injection, Intravenous injection) | Frequency of injection (Every week, Every other week, Every month, Every 3 months) | Ability to reduce pain and joint inflammation (No change, 25% reduction, 50% reduction, 75% reduction) | Ability to conserve physical function (0%, 20%, 40%, 60%) | Low risk for cancer or infection (yes, no) | Local adverse effect (15 individuals from 100 individuals, 40 individuals from 100 individuals) | Onset of action (till 1 month, 1 to 3 months) | Out-of-pocket cost (20$, 40$, 60$) |  |

Notes: Levels in parentheses.

**References**

1. Nazari A, Lopez-Valcarcel BG, Najafi S. Preferences of Patients With HR+ & HER2- Breast Cancer Regarding Hormonal and Targeted Therapies in the First Line of Their Metastatic Stage: A Discrete Choice Experiment. Value in Health Regional Issues. 2021;25: 7–14. doi:10.1016/j.vhri.2020.10.002

2. Ehsani-Chimeh E, Keikavoosi-Arani L, Zohrevandi B, Asghari A, Rad EH. Willingness to Pay in Choosing Pre-hospital Emergency Services in Iran: A Population-Based Discrete Choice Experiment. Health Technology Assessment in Action. 2024;8. doi:10.18502/htaa.v8i2.15629

3. Mehranfard Z, Mozayani A, Arani AA, Agheli L. Estimating Individuals’ Willingness to Pay to Avoid Corona Disease’s Social Consequences. Iranian Economic Review. 2024;28: 680–694. doi:10.22059/ier.2022.90124

4. Doroh V, Delavari A, Yaseri M, Sefiddashti S, Sari A. Preferences for Colorectal Cancer Screening Tests: Differences Between Insured and Uninsured Beneficiaries of Iranian Health Transformation Plan. HEALTH SCOPE. 2018;7. doi:10.5812/jhealthscope.63213

5. Ansari F, Rafiei S, Kakemam E, Amerzadeh M, Ahadinezhad B. Eliciting preference for private health services among patients in Iran: evidence from a discrete choice experiment. International Journal of Human Rights in Healthcare. 2023;16: 316–326. doi:10.1108/IJHRH-06-2021-0137

6. Zartab S, Nikfar S, Jamshidi A, Toroski M, Varahrami V, Fatemi B, et al. How do patients value features of biological medicine in rheumatoid arthritis? A discrete choice experiment. Expert Rev Pharmacoecon Outcomes Res. 2023;23: 701–708. doi:10.1080/14737167.2023.2210836

7. Sarikhani Y, Ghahramani S, Edirippulige S, Fujisawa Y, Bambling M, Bastani P. What do Iranian physicians value most when choosing a specialty? Evidence from a discrete choice experiment. Cost Eff Resour Alloc. 2022;20: 23. doi:10.1186/s12962-022-00358-z

8. Sargazi N, Takian A, Yaseri M, Daroudi R, Ghanbari Motlagh A, Nahvijou A, et al. Mothers’ preferences and willingness-to-pay for human papillomavirus vaccines in Iran: A discrete choice experiment study. Prev Med Rep. 2021;23: 101438. doi:10.1016/j.pmedr.2021.101438

9. Ranjbar M, Bazyar M, Pahlevanshamsi F, Angell B, Assefa Y. What do Iranians value most when choosing a hospital? Evidence from a discrete choice experiment. PLoS One. 2024;19: e0293241. doi:10.1371/journal.pone.0293241

10. Ranjbar Ezatabadi M, Rashidian A, Shariati M, Rahimi Foroushani A, Akbari Sari A. Using Conjoint Analysis to Elicit GPs’ Preferences for Family Physician Contracts: A Case Study in Iran. Iran Red Crescent Med J. 2016;18: e29194. doi:10.5812/ircmj.29194

11. Ramezani Doroh V, Delavari A, Yaseri M, Emamgholipour Sefiddashti S, Akbarisari A. Preferences of Iranian average risk population for colorectal cancer screening tests. Int J Health Care Qual Assur. 2019;32: 677–687. doi:10.1108/ijhcqa-08-2017-0151

12. Rahimi F, Rasekh HR, Abbasian E, Peiravian F, Etemadifar M, Ashtari F, et al. Patient preferences for Interferon-beta in Iran: A discrete choice experiment. PLoS One. 2018;13: e0193090. doi:10.1371/journal.pone.0193090

13. Rafiei S, Arab M, Rashidian A, Mahmoudi M, Rahimi-Movaghar V. Policy interventions to improve rural retention among neurosurgeons in Iran: A discrete choice experiment. Iran J Neurol. 2015;14: 211–8.

14. Parvizi S, Mehrara M, Taiebnia A. Investigating preferences of the covid-19 vaccine among individuals in Iran: Discrete choice experiment analysis. Health Sci Rep. 2023;6: e1332. doi:10.1002/hsr2.1332

15. Nargesi DA, Hajizadeh M, Pakdel MJ, Gheysvandi E, Rad EH. Preferences of Iranians to select the emergency department physician at the time of service delivery. BMC Health Serv Res. 2021;21: 1155. doi:10.1186/s12913-021-07183-9

16. Moradi K, Khosravi K, Jalali A, Rezaei S. From classroom to workforce: a discrete choice analysis of Iranian nursing students’ job preferences. BMC Med Educ. 2025;25: 15. doi:10.1186/s12909-024-06612-7

17. Kazemi-Karyani A, Ramezani-Doroh V, Khosravi F, Miankali ZS, Soltani S, Soofi M, et al. Eliciting preferences of patients about the quality of hospital services in the west of Iran using discrete choice experiment analysis. Cost Eff Resour Alloc. 2021;19: 65. doi:10.1186/s12962-021-00319-y

18. Kazemi Karyani A, Karami Matin B, Malekian P, Moradi Rotvandi D, Amini S, Delavari S, et al. Preferences of Medical Sciences Students for Work Contracts in Deprived Areas of Iran: A Discrete Choice Experiment Analysis. Risk Manag Healthc Policy. 2020;13: 927–939. doi:10.2147/rmhp.S259267

19. Kazemi Karyani A, Akbari Sari A, Woldemichael A. Eliciting Preferences for Health Insurance in Iran Using Discrete Choice Experiment Analysis. Int J Health Policy Manag. 2019;8: 488–497. doi:10.15171/ijhpm.2019.29

20. Jouyani Y, Hadiyan M, Salehi M, Souri A. Using discrete choice model to elicit preference for health-care priority setting. J Educ Health Promot. 2019;8: 117. doi:10.4103/jehp.jehp_404_18

21. Jouyani Y, Bahrampour M, Barouni M, Dehnavieh R. Patient preferences for hospital quality: case study of iran. Iran Red Crescent Med J. 2013;15: 804–8. doi:10.5812/ircmj.12851

22. Homaie Rad E, Hajizadeh M, Yazdi-Feyzabadi V, Delavari S, Mohtasham-Amiri Z. How Much Money Should be Paid for a Patient to Isolate During the COVID-19 Outbreak? A Discrete Choice Experiment in Iran. Appl Health Econ Health Policy. 2021;19: 709–719. doi:10.1007/s40258-021-00671-3

23. Homaie Rad E, Hajizadeh M, Rajabpour M, Mohtasham-Amiri Z, Rahbar-Taramsari M, Bahador F, et al. Preferences of Iranian medical students for selecting the compulsory service plan packages: A discrete choice experiment. Health Sci Rep. 2024;7: e2213. doi:10.1002/hsr2.2213

24. Haddadfar A, Emamgholipour S, Razani M, Salehnejad MH. Nurses’ job preferences for working in deprived areas in Tehran: a discrete choice experiment. Hum Resour Health. 2023;21: 91. doi:10.1186/s12960-023-00875-9

25. Bahrampour M, Bahrampour A, Amiresmaili M, Barouni M. Hospital service quality - patient preferences - a discrete choice experiment. Int J Health Care Qual Assur. 2018;31: 676–683. doi:10.1108/ijhcqa-01-2017-0006

26. Darrudi A, Daroudi R, Yunesian M, Akbari Sari A. Public Preferences and Willingness to Pay for a COVID-19 Vaccine in Iran: A Discrete Choice Experiment. Pharmacoecon Open. 2022;6: 669–679. doi:10.1007/s41669-022-00359-x

27. Afsharmanesh G, Rahimi F, Zarei L, Peiravian F, Mehralian G. Public and decision-maker stated preferences for pharmaceutical subsidy decisions in Iran: an application of the discrete choice experiment. J Pharm Policy Pract. 2021;14: 74. doi:10.1186/s40545-021-00365-0

28. Afshari S, Daroudi R, Goudarzi R, Mahboub-Ahari A, Yaseri M, Sari AA, et al. A national survey of Iranian general population to estimate a value set for the EQ-5D-5L. Qual Life Res. 2023;32: 2079–2087. doi:10.1007/s11136-023-03378-1

29. Delpasand M, Olyaaeemanesh A, Jaafaripooyan E, Abdollahiasl A, Davari M, Kazemi Karyani A. Eliciting the public preferences for pharmaceutical subsidy in Iran: a discrete choice experiment study. J Pharm Policy Pract. 2021;14: 59. doi:10.1186/s40545-021-00345-4
